# Supplementary material for: The causal role of circulating inflammatory markers in osteoporosis: a bidirectional Mendelian randomized study
Source: Front Immunol. 2024 Jul 18;15:1412298. doi: 10.3389/fimmu.2024.1412298 (PMC11291241; doi:10.3389/fimmu.2024.1412298)
Supplement: Supplementary file 1 [file DataSheet_1.zip › supplementary material/OSTEOPOROSIS-CIM/GCST90274774.tsv.gz_leave_one_out.pdf]

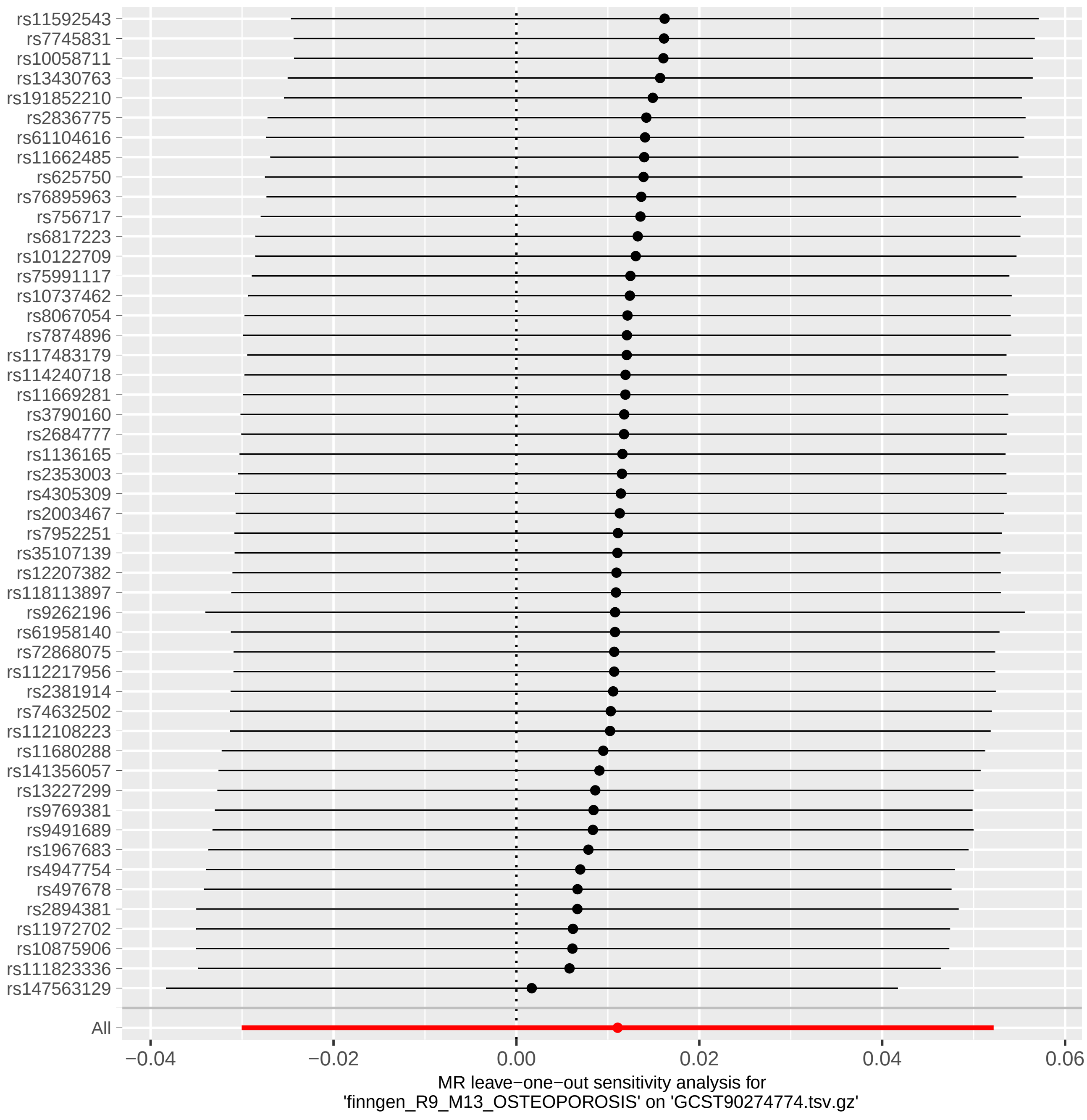
rs11592543
rs7745831
rs10058711
rs13430763
rs191852210
rs2836775
rs61104616
rs11662485
rs625750
rs76895963
rs756717
rs6817223
rs10122709
rs75991117
rs10737462
rs8067054
rs7874896
rs117483179
rs114240718
rs11669281
rs3790160
rs2684777
rs1136165
rs2353003
rs4305309
rs2003467
rs7952251
rs35107139
rs12207382
rs118113897
rs9262196
rs61958140
rs72868075
rs112217956
rs2381914
rs74632502
rs112108223
rs11680288
rs141356057
rs13227299
rs9769381
rs9491689
rs1967683
rs4947754
rs497678
rs2894381
rs11972702
rs10875906
rs111823336
rs147563129
All
−0.04
−0.02
0.00
0.02
0.04
0.06
MR leave−one−out sensitivity analysis for
'finngen_R9_M13_OSTEOPOROSIS' on 'GCST90274774.tsv.gz'
